# Determination of Perfluorooctanoic Acid (PFOA) in the Indoor Dust Matter of the Sicily (Italy) Area: Analysis and Exposure Evaluations

**DOI:** 10.3390/toxics12010028

**Published:** 2023-12-28

**Authors:** Salvatore Barreca, Michele Marco Mizio Mancuso, Daniel Sacristán, Andrea Pace, Dario Savoca, Santino Orecchio

**Affiliations:** 1 Department of Chemical Sciences, University of Catania, Viale A. Doria 6, 95100 Catania, Italy; 2 Department of Biological, Chemical and Pharmaceutical Sciences and Technologies, University of Palermo (STEBICEF), Viale Delle Scienze, Ed. 17, 90100 Palermo, Italyandrea.pace@unipa.it (A.P.); dario.savoca@unipa.it (D.S.); santino.orecchio@unipa.it (S.O.); 3 Department of Plant Biology, University of Valencia Córdoba, 46100 Valencia, Spain; daniel.sacristan@uv.es; 4 Department of Soil and Environmental Quality Department, Centro de Investigaciones sobre Desertificación-CIDE (CSIC-Universitat de València-Generalitat Valenciana), Carretera Moncada-Náquera km 4.5, 46113 Valencia, Spain; 5 NBFC—National Biodiversity Future Center, 90123 Palermo, Italy

**Keywords:** UHPLC-QTOF-MS/MS, PFOA quantification, indoor dust analysis, PFOA Exposure Index

## Abstract

Perfluorooctanoic acid (PFOA) in environmental matrices is increasingly being studied due to its environmental persistence, global occurrence, bioaccumulation, and associated human health risks. Some indoor environments can significantly impact the health of occupants due to pollutants in indoor air and household dust. To investigate the potential exposure of individuals to PFOA in specific confined environments, this study reports an analytical method and results concerning the determination of PFOA in household dust, used as a passive sampler. To the best of our knowledge, this paper represents one of the first studies concerning PFOA concentrations in indoor dust collected in the south of Italy, within the European region. A total of twenty-three dust samples were collected from two different areas of Sicily (Palermo and Milena), extracted, and analyzed by an UHPLC-QTOF-MS/MS system. Finally, PFOA exposure was estimated using a new index (Indoor PFOA Exposure Index, IPEX) that incorporates the PFOA levels in dust, exposure time, and the correlation between the PFOA in dust and blood. It was then compared across four different exposure groups, revealing that PFOA exposure for people working in chemistry laboratories was evaluated to be ten times higher than the exposure for homemakers.

## 1. Introduction

In recent years, various pollutants have been detected in different environmental matrices, including soils, sediments, water, air, and dust [1,2,3,4,5,6]. Some of these pollutants, such as perfluorocarbons or polychlorinated biphenyls, have been classified as persistent pollutants [7,8].

The term PFAS is generally used to refer to a group of anthropogenic organic compounds composed of poly- and perfluoroalkyl compounds characterized by a hydrophilic group and a fluorinated alkyl chain, typically ranging from C4 to C12 [9,10]. The C–F bond imparts specific physicochemical properties to this class of substances [11]. Due to these characteristics, PFAS have multiple applications and are widely used in everyday products, such as paints, polishes, adhesives, textiles, waxes, and stain/water/grease repellents in carpets and clothing. They are also found in kitchen utensils, like in non-stick coatings [12,13,14]. Unfortunately, (C–F) chemical bonds make PFAS very stable and they do not degrade when exposed to heat, acids, or oxidation substances, nor do they degrade naturally [15,16]. Because of these strong properties, PFAS are classified as highly persistent pollutants [17]. Moreover, their resistance to chemical, biological, and physical factors suggests that the human impact on exposure from materials and consumer products is generally lower than that from drinking water and/or contaminated food, such as fish [18,19,20,21,22]. PFAS can accumulate in the human body and, as reported in the literature, their levels decrease slowly over time [23]. Furthermore, PFAS may impact the immune system, fertility, fetus development, and cholesterol levels and increase the risk of cancer [24,25]. Consequently, due to their negative impact on health, PFAS have recently attracted attention from the scientific community, leading to specific regulations on the use of these compounds, especially for perfluorooctane sulfonate (PFOS) and perfluorooctanoic acid (PFOA), which have been added to the list of “dangerous and priority” substances to be monitored in water bodies [26].

Although PFAS include thousands of chemicals, environmental studies primarily focus on perfluorooctanoic acid (PFOA) and perfluorooctanesulfonic acid (PFOS), with PFOA being the most widely used and detected in several environmental matrices [19,27,28,29]. PFOA is persistent in the environment and bioaccumulates in the food chain. The United States Environmental Protection Agency (US EPA) has proposed a value of 400 ng L^−1^ as the Environmental Quality Standard (EQS) for drinking water [30]. It is essential to note that PFOA has been detected and quantified in indoor dust from various geographic areas, such as the Americas and Europe, with statistically significant correlations found between PFOS and PFOA concentrations in dust samples [31].

Moreover, considering that PFOA, classified as a carcinogenic substance by the International Agency for Research on Cancer (IARC by WHO), can increase the chances of kidney and testicular cancer, it has been one of the most investigated PFAS. Generally, indoor air quality is investigated using dedicated automatic instruments that sample large air volumes. In this context, a realistic difficulty in analyzing environmental pollutants is the very low concentration; indoor dust can be used as a passive sampler to assess the quality of the indoor environment and estimate human and child exposure to pollutants [31,32,33,34,35].

Furthermore, dust can be a significant source of contamination for children and adults. For example, children may ingest settled dust by playing on the floor indoors. For these reasons, possible risk exposure can be assessed by analyzing indoor dust. Settled dust is efficient for determining exposure, providing information on the average variation in the space and time of contaminants in the area under investigation. In this context, a recent study reported the bioavailability of PFOA in the lungs following inhalation exposure to house dust collected from residential homes. Additionally, it was reported that the bioavailability of PFOA in the lungs, following inhalation exposure to house dust collected from residential homes, is almost four times higher in blood at 3 h post-exposure compared to oral gavage exposure [36]. Notably, several authors have reported a strong correlation between PFOA and PFOS not only in indoor dust samples but also in biological samples and environmental media. Strong correlations were found in offices (r = 0.65), homes (r = 0.83), and vehicles (r = 0.90) [37,38]. Furthermore, various studies have reported similar correlations in house dust between PFOS and PFOA, with r-values ranging from 0.75 to 0.86 [31,34]. In this context, preliminary exposure to PFAS can be investigated by determining either PFOA or PFOS in indoor dust samples.

The aim of this work is to extend the Ultra-High-Performance Liquid Chromatography-Quadrupole Time-Of-Flight Mass Spectrometry (UHPLC-QTOF-MS) method to quantify PFOA (used as a model pollutant for PFAS) in indoor settled dust (used as a passive sampler) collected in the Sicilian area. This is to evaluate the distribution and concentrations inside common indoor environments. Indoor dust samples were collected from various buildings located in Sicily, a region in southern Italy considered both tourist and commercial with a population of about 5 million. While many studies report PFOA concentrations in several states, unfortunately, most of these studies have focused on the United States of America and northern European areas.

In the present study authors investigated two different areas: one highly anthropized (Palermo), analyzing both houses and the chemistry laboratories of Palermo University, and another area (Milena) that is less anthropized, also analyzing both houses and the chemistry laboratories of Palermo University. To the best of our knowledge, this research represents the first evidence and evaluation of the presence of PFOA in indoor dust samples in Sicily.

## 2. Materials and Methods

### 2.1. Sampling

Indoor dust samples were obtained from buildings located in two areas of Sicily (Palermo and the city of Milena). Sampling procedures followed similar methods as those reported in the literature [6]. In summary, samples were carefully collected from household surfaces (shelves, furniture, etc.) using a brush, transferred to aluminum foil, identified by a unique codex, placed in an individual glass vial to avoid cross-contamination, and stored at room temperature. Sampling covered exposed surfaces, such as the floor, bookshelves, counters, moldings, and lampshades; additionally, dirt, sand, and gravel were not collected. In the laboratory, a portion of each sample was weighed and stored in dark conditions until extraction. A total of 23 samples were collected; Table 1 provides information on the sample codex, sampling area, and sample weight.

Specifically, three samples were collected from a country home in the town of Milena (Group a), five samples were collected from another house in the city of Milena (Group b), five samples were collected in a laboratory at Palermo University where PFOA was used as a precursor for organic chemistry synthesis (Group c), seven samples were collected from the bedrooms of a student university residence situated in Palermo city near an anthropized area (Group d), and three samples were collected from another laboratory at Palermo University generally used for environmental and analytical chemistry analyses and procedures (Group e).

Palermo is the largest and most urbanized city in Sicily, Italy, with an annual mean temperature of 23 °C and an annual relative humidity of 71.5%. Indoor environments in Palermo are typically ventilated through windows and, in some cases, through air-conditioning systems.

Milena, on the other hand, is a small town in Sicily with low urbanization, characterized by an annual mean temperature of 21 °C and an annual relative humidity of 70%. Despite the differences in size and urbanization, the climatic conditions in Palermo and Milena are comparable. In both locations, ventilation in indoor environments is generally achieved through windows, occasionally supplemented by air-conditioning systems.

For these similar climatic conditions, indoor environments in Palermo and Milena can be considered comparable.

### 2.2. Materials, Equipment, and Software

Methanol of LC-MS purity grade (from Honeywell, Charlotte, NC, USA) was utilized for extractions and analyses. LC-MS grade water (from PanReac AppliChem, Chicago, IL, USA) was used for HPLC-MS analyses preconditioning or washing procedures. Ammonium acetate (from Aldrich, St. Louis, MO, USA) served as an additive for HPLC eluents during analyses. Sodium sulfate, purchased from Aldrich, was used in the filtration process. The analytical standard of perfluorooctanoic acid (PFOA), with a purity grade exceeding 98% and acquired from Aldrich, was employed for LC-MS calibration curves and for preparing spiking solutions. PFOA-free polypropylene micropipette tips were used for small-volume withdrawals to prevent any PFOA contamination. The equipment used for sampling and extraction procedures was thoroughly washed with methanol to avoid contamination. LC-MS analyses were conducted using a 6540 UHD Accurate-Mass Q-TOF LC-MS/MS system (Agilent Technologies, Santa Clara, CA, USA) equipped with an Electron Spray Ionization source type Dual AJS ESI, operating in negative ionization mode.

### 2.3. Sample Extraction and Method Optimization

Since certified reference material containing PFOA in dust is not readily available, the authors conducted recovery experiments on spiked dust samples to validate the accuracy and precision of the analytical procedure corresponding to the low calibration level of the calibration curve (PFOA in water at 100 ng L^−1^). After PFOA extraction (confirmed by LC-MS/MS analysis to be absent in the blank), a known amount of PFOA was added to the purified blank sample. The best recovery performances (percentage recoveries of 85 ± 8%) were achieved with three extractions using methanol. The method’s limit of quantification (LOQ) was evaluated and calculated as the concentration corresponding to a signal-to-noise ratio ≥ 10, as per IUPAC criteria (LOQ corresponding to 4.0 ng g^−1^).

For extraction procedures, approximately 100 mg of each dust sample was weighed on an analytical balance, transferred to a flask, and 25 mL of methanol was added. The samples were then placed onto the ultrasonic bath extraction system three times. The extracted material was filtered through sodium sulfate to remove adsorbed water and the eluate was collected. This extraction process was repeated three times and the three different fractions were combined and evaporated. After evaporation, the sample was reconstituted to 5 mL with methanol in a volumetric flask and prepared for analysis.

Analyses were carried out using an Agilent Infinity 1260 HPLC system connected to an Agilent Technologies ESI-QTOF UHD 6540 MS/MS detector, operating in the negative ion-monitoring mode. A volume of 15 μL of sample from extracts or standard solutions was injected, using a 50 μL syringe, into a certified loop of 10 μL. Separations were performed on a Phenomenex Poroshell EC-C18 3.0 × 50 mm 2.7 μm column, using a mixture of water containing 4 mM ammonium acetate and methanol with a fixed flow of 0.4 mL/min.

The elution gradient was as follows: from Water/MeOH 50/50 (*vol*/*vol*) to Water/MeOH 5/95 in 2 min, to Water/MeOH 2/98 in 1 min, and maintaining elution with Water/MeOH 2/98 for an additional 4 min before returning to Water/MeOH 2/98 in 1 min. Subsequently, Water/MeOH 95/5 was used for 2 min, followed by maintaining elution with Water/MeOH 95/5 for an additional 5 min before returning to the initial conditions over 3 min. Data were analyzed using the Agilent Mass Hunter Workstation software (Version 3.0). PFOA was identified through MS spectrum monitoring ([M-H] - ion at 412.9664 ± 0.0005 Dalton) and targeted MS/MS (monitoring the 412.966 ± 0.002 Dalton ≥ 368.976 ± 0.002 Dalton fragmentation). The first transition (412.966) was used for quantification while the second transition (368.976) was used for confirmation. PFOA was not detected in the blank (MeOH) analyses conducted every two runs, confirming the absence of cross-contamination and the high quality of the analyses.

Analytical batches comprised method blanks, solvent blanks, QA/QC samples, calibration curve samples, and unknown samples. Quality checks were performed by analyzing a PFOA standard solution at 10 ng L^−1^ every ten samples. Precision ranged between 2% and 10.2% and accuracy was between 83.1% and 107.0% for all items in the quality control process. All samples underwent the same extraction procedure and analytical conditions.

## 3. Results and Discussion

### 3.1. Sample Analysis and Evaluation

As noted in previous studies [34,35], this investigation recorded a high detection frequency of perfluorooctanoic acid (PFOA) in the majority of the analyzed samples. Specifically, PFOA was present in 96% of the samples, with concentrations ranging from 29.4 to 3385 ng g^−1^ (mean: 443.3 ng g^−1^; see Table 2).

The highest concentrations of PFOA were identified in Samples 4c and 5d (3385 and 2292 ng g^−1^, respectively), both collected from an organic chemistry laboratory (I). This laboratory frequently utilizes PFOA as a reagent in organic synthesis.

Significant variations in perfluorooctanoic acid (PFOA) concentrations were observed among different sampling cities and locations. Specifically, in the dust samples collected in Milena, PFOAs were detected in 87% of the analyzed samples, with concentrations ranging from below the limit of quantification (LOQ) in one sample to 456 ng g^−1^, averaging at 158 ng g^−1^. On the other hand, in the dust collected in the city of Palermo, the analyte was present and quantified in 100% of the analyzed samples. In this case, concentrations ranged from 29.4 to 3385 ng g^−1^, with a mean of 576 ng g^−1^. Notably, the lowest average concentration (42.3 ng g^−1^) (refer to Table 3) was identified in samples obtained from a country house in Milena, an area with a low anthropization impact, especially in rural settings. It is intriguing to observe that the mean concentrations in different macroenvironments (laboratory and house) are notably high, differing by a factor of 36, and this difference diminishes when considering microenvironments. Specifically, the mean concentration difference for samples collected in Laboratory I and Laboratory II is 18 times while the mean concentration difference for samples collected in the Milena house is 4.6 times.

**Figure 1:**
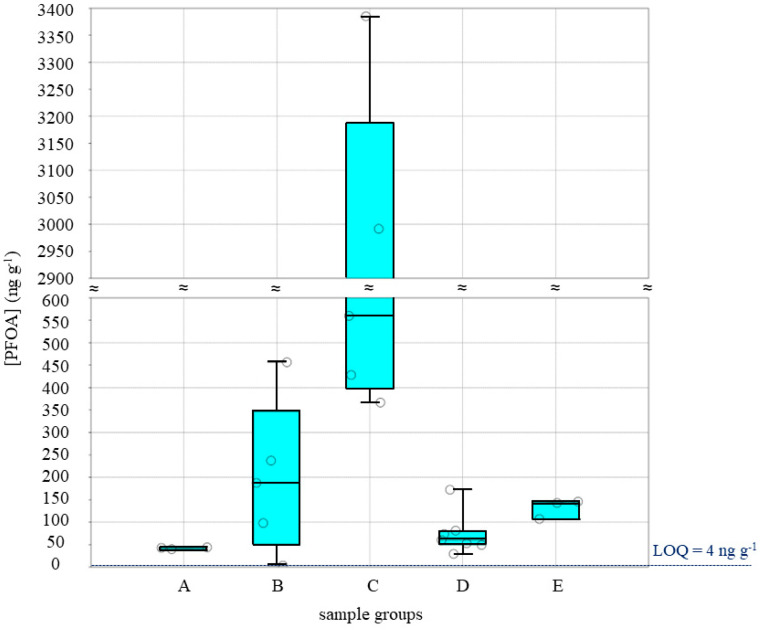
Box and jitter plots showing the PFOA concentrations [PFOA] expressed in ng g^−1^ found for each of the five class groups belonging to the different sampling areas. The 25–75 percentiles are drawn using a box; the minimum and maximum are shown at the end of the thin lines (whiskers); the median is marked as a horizontal line within the box plot; the LOQ value is shown at the bottom as a horizontal dashed line.

A statistical summary concerning the five class groups as the graphic is reported in Figure 1.

In Palermo, notable concentrations of perfluorooctanoic acid (PFOA) were observed in the dust samples collected from the laboratory building in category I (Samples from 1c to 5c). Specifically, when compared to other building categories, the levels of PFOA were significantly higher in this category. PFOA concentrations were found to be comparable between dust samples collected in indoor houses in Milena and Palermo buildings. However, among the Milena building groups, Group a exhibited the lowest concentrations.

Nevertheless, the levels of PFOA analyzed in house dust samples are in the same order of magnitude when compared with other published data from studies conducted throughout Europe, the United States of America, and Japan [31]. For instance, PFOA concentrations in Swedish apartments’ dust ranged from 17 to 850 ng g^−1^; in the United States of America, PFOA was quantified in the range of 10 to 1960 ng g^−1^; and in Japan, the levels of PFOA ranged from 70 to 3700 ng g^−1^.

In this context, the data reported in this study align with the existing literature and affirm the widespread use of PFOA in numerous everyday products. Furthermore, considering that PFOA in office worker serum is more strongly associated with other per- and polyfluoroalkyl substances (PFAS) levels in office air than in dust, the authors have developed a new Indoor PFOA Exposure Index (IPEX). This index aims to assess PFOA exposure during indoor occupancy, expressed as ng g^−1^ h, for various situations both at work and during in-house occupancy.

### 3.2. Evaluation of PFOA Exposure

To assess and differentiate exposure to contaminants in this case study, perfluorooctanoic acid (PFOA) concentrations found in dust samples collected from various environments were utilized to estimate exposure for two distinct groups—chemist workers and household individuals—and two subgroups (chemist workers considering two different laboratories and household individuals from two different cities). In summary, the group comparison was employed to evaluate PFOA indoor exposure among different occupational groups while the subgroup comparison was utilized to assess PFOA indoor exposure for individuals originating from different cities.

Daily PFOA exposure from dust was calculated by considering the PFOA concentration in indoor dust samples, the amount of personal time spent in indoor environments, and the correlation between PFOA concentration in dust samples and PFOA levels in the blood.

Specifically, the authors considered the following factors:

Chemists typically spend 18 h in indoor environments, comprising 8 h in the laboratory and 10 h in indoor households;

Household individuals typically spend 18 h in indoor environments, specifically within their homes;

The peak concentration of PFOA (PFOA Cmax) in blood samples occurs 3 h post-exposure and the Cmax value is 100 times lower than PFOA levels detected in indoor dust [36].

Based on these considerations, an Indoor PFOA Exposure Index (IPEX), expressed as ng g^−1^ h, was calculated as follows:

$$\mathrm{IPEX}\;=\;\frac{\left[\mathrm{PFOA}\;\mathrm{dust}\right]\times \left[\mathrm{indoor}\;\mathrm{hours}\right]}{100}$$

where:

- PFOA dust is PFOA concentration in dust samples expressed as ng g^−1^;
- Indoor hours are time (in hours) that people spent in indoor environments;
- The 100 value is the correlation factor concerning PFOA levels from dust to blood samples.

The calculated Indoor PFOA Exposure Index (*IPEX*) for each group and subgroup considered are reported in Table 4.

Referring to the values presented in Table 4, the Indoor PFOA Exposure Index (IPEX) value for chemists in Palermo working at Laboratory I, where PFOA is commonly used as a precursor for organic synthesis, is observed to be 10 times higher than the exposure of housewomen/housemen in Palermo. However, the exposure for a chemist working in a laboratory where PFOA is not used as a precursor falls within the same order of magnitude and is comparable to other calculated IPEX values.

## 4. Conclusions

To the best of our knowledge, this study represents the first investigation and evaluation of perfluorooctanoic acid (PFOA) levels in indoor dust conducted in the southern region of Italy.

Our research provides valuable insights into the concentrations of PFOA in the indoor dust of chemistry laboratories and homes collected in Sicily, an Italian region, offering an assessment of exposure to PFOA—an important emerging pollutant selected as a model for compounds of per- and polyfluoroalkyl substances (PFAS).

The use of ultra-high-performance liquid chromatography-quadrupole time-of-flight mass spectrometry proved particularly suitable for our purposes due to its wide linearity range. Using the same calibration line, it allowed the analysis of solutions with concentrations varying from a few ng g^−1^ to several ppm, covering a range of several orders of magnitude. This capability was especially beneficial for analyzing solutions derived from environmental matrices, like indoor dust, characterized by significant compositional heterogeneity. Considering the standard deviation values of found concentrations, we assert that the method provides accurate results, making it suitable for determining PFOA concentrations.

PFOA concentrations were determined in twenty-three home dust samples collected from two different houses in Sicily. Our investigation sheds light on PFOA concentrations in indoor dust in the southern region of Italy. PFOA was significantly quantified in most samples, with 96% of them showing concentrations ranging from 29.4 to 3385 ng g^−1^. The highest average concentrations (1546 ng g^−1^) were detected in an indoor dust sample collected in an organic chemistry laboratory that used PFOA as a precursor in organic synthesis.

This study underscores that PFOA exposure for individuals working in specific chemistry laboratories is ten times higher than the exposure for housewomen/housemen. The reported results align with the previous literature data, emphasizing the ubiquitous presence of PFOA in indoor environments and suggesting higher levels in anthropized areas. Furthermore, the results indicate that PFOA levels in chemistry laboratories are higher than in homes.

Based on the information provided in this research article, we can conclude that PFOA is a detectable pollutant in dust samples in the Sicily region. This study represents the first assessment of PFOA levels in dust samples collected in Sicily, Italy.

In conclusion, this research demonstrates that PFOA is present in indoor dust in most sampled environments and that dust can represent an important route of exposure to compounds of PFAS.

## Figures and Tables

**Table 1:** Codex sample, sampling area, and sample weight concerning indoor dust samples.

Codex Sample	Sampling Area	Sample Weight (g)
1a	Milena country house	0.14935
2a	Milena country house	0.14907
3a	Milena country house	0.14897
1b	Milena city house	0.13941
2b	Milena city house	0.12813
3b	Milena city house	0.14631
4b	Milena city house	0.13095
5b	Milena city house	0.12751
1c	Palermo (University. STEBICEF) Laboratory I	0.12495
2c	Palermo (University. STEBICEF) Laboratory I	0.13510
3c	Palermo (University. STEBICEF) Laboratory I	0.14309
4c	Palermo (University. STEBICEF) Laboratory I	0.13178
5c	Palermo (University. STEBICEF) Laboratory I	0.14626
1d	Palermo (Santi Romano. student University residence)	0.09811
2d	Palermo (Santi Romano. student University residence)	0.10196
3d	Palermo (Santi Romano. student University residence)	0.10302
4d	Palermo (Santi Romano. student University residence)	0.11484
5d	Palermo (Santi Romano. student University residence)	0.09736
6d	Palermo (Santi Romano. student University residence)	0.10929
7d	Palermo (Santi Romano. student University residence)	0.10216
1e	Palermo (University. STEBICEF) Laboratory II	0.15313
2e	Palermo (University. STEBICEF) Laboratory II	0.17358
3e	Palermo (University. STEBICEF) Laboratory II	0.16489

**Table 2:** Sampling city, codex sample identification, PFOA concentration (ng g^−1^), and mean (ng g^−1^) in house dust samples.

City of Sampling	Codex Sample	PFOA ng g^−1^
Milena country house	1a	44.01
Milena country house	2a	42.92
Milena country house	3a	39.82
Milena city house	1b	237.21
Milena city house	2b	<LOQ
Milena city house	3b	98.05
Milena city house	4b	456.45
Milena city house	5b	187.53
Palermo (University. STEBICEF) Laboratory I	1c	559.83
Palermo (University. STEBICEF) Laboratory I	2c	428.02
Palermo (University. STEBICEF) Laboratory I	3c	366.68
Palermo (University. STEBICEF) Laboratory I	4c	3385.24
Palermo (University. STEBICEF) Laboratory I	5c	2991.56
Palermo (Santi Romano. student University residence)	1d	60.31
Palermo (Santi Romano. student University residence)	2d	73.72
Palermo (Santi Romano. student University residence)	3d	29.38
Palermo (Santi Romano. student University residence)	4d	81.26
Palermo (Santi Romano. student University residence)	5d	172.3
Palermo (Santi Romano. student University residence)	6d	52.93
Palermo (Santi Romano. student University residence)	7d	49.19
Palermo (University. STEBICEF) Laboratory II	1e	145.75
Palermo (University. STEBICEF) Laboratory II	2e	142.92
Palermo (University. STEBICEF) Laboratory II	3e	107.22
PFOA average in Palermo samples		157.99
PFOA average in Milena samples		576.42
Total average		444.3

**Table 3:** PFOA value in terms of min–max range, sum, mean, and variance considering different sampling groups.

	Group a	Group b	Group c	Group d	Group e
N samples	3	5	5	7	3
Min (ng g^−1^)	39.8	2.5	367	29.4	107
Max (ng g^−1^)	44.0	456	3385	172	146
Sum (ng g^−1^)	127	982	7731	519	395
Mean (ng g^−1^)	42.3	196	1546	74.1	132
Variance	4.7257	29,160.01	2.271,413	2158.891	461.1766

**Table 4:** Indoor PFOA Exposure Index (*IPEX*) *evaluation concerning groups and subgroups*.

Group	*IPEX* *Indoor Laboratory* ng g^−1^ day	*IPEX* *Indoor House* ng g^−1^ day	*IPEX* *Total Indoor* ng g^−1^ day
Chemist Palermo from laboratory I	124	7.4	131.4
Chemist Palermo from laboratory II	10.6	7.4	18
Housewoman/Houseman from Milena rural house	---	7.6	7.6
Housewoman/Houseman from Palermo	---	13.3	13.3

## Data Availability

The original data presented in the study are included in the article; further inquiries can be directed to the corresponding author.
